# Mental health problems and associated factors in secondary school students from Foshan, Guangdong, China

**DOI:** 10.3389/fpsyg.2025.1626236

**Published:** 2025-09-19

**Authors:** Yaodong Li, Ting Peng, Junxian Liu, Huanyu Xu, Jie Lian, Jiarong Lei, Zaiping Huang, Jieping Lin, Qingmei Zheng, Cai Zhao, Yu Huang, Wen Wang, Guang Yang, Rongdi Liang, Yu Dai, Xiaonian Luo

**Affiliations:** ^1^The Fourth People’s Hospital of Shunde (Shunde Wu Zhongpei Memorial Hospital), Foshan, China; ^2^Southwest Campus of Shunde No.1 High School, Foshan, China

**Keywords:** secondary school children, mental health, associated factors, MMHI-60, SRBES

## Abstract

**Background:**

Secondary school students are in a relatively sensitive adolescent period, which is critical for mental development. The present study aimed to examine the mental health status and associated factors in secondary school students.

**Methods:**

Secondary school students were enrolled from all the five secondary schools in Shunde District, Guangdong. The Mental Health Inventory of Middle School Students (MMHI-60), School Refusal Behavior Evaluation Scale (SRBES), School Refusal Assessment Scale for Children (SRAS-C), Revised Chinese Internet Addiction Scale (CIAS-R), Pittsburgh Sleep Quality Index (PSQI), Adolescent Suicide Tendency Scale (ASTS), Connor-Davidson Resilience Scale (CD-RISC), and Self-esteem scale (SES) were used to assess mental health problems and determine associated factors in secondary school students.

**Results:**

A total of 8,013 secondary school students were included. The prevalence of abnormal mental health was 22.4% by MMHI-60, 19.7% by SRBES, 5.4% by CIAS-R and 20.3% by ASTS, respectively. The scores of CD-RISC and SES were positively correlated with each other, and negatively correlated with the scores of SRBES, CIAS, PSQI, ASTS, and MMHI-60 (*p* < 0.05). The scores of SRBES, CIAS, PSQI, ASTS, and MMHI-60 were positively correlated with each other (*p* < 0.05). There were 62 students (0.8%) who showed abnormal mental problems suggested by all four scales of SRBES, CIAS-R, MMHI-60, and ASTS. Girls had significantly higher scores of SRBES, SRAS-C, PSQI, ASTS, CIAS-R, and MMHI-60 but significantly lower scores of SES and CD-RISC than boys (*p* < 0.05). Additionally, economic status, father’s education, mother’s education and grade rank were also associated with the above eight scales (*p* < 0.05).

**Conclusion:**

Our study demonstrated the mental health problems in secondary school students and associated factors, which are essential for developing targeted interventions and policies to support the mental well-being of this vulnerable population.

## Introduction

Adolescence is a pivotal stage in human development, characterized by significant physical, emotional and cognitive changes. This period is marked by heightened sensitivity to external influences and internal pressures, which can significantly impact the mental health of young individuals (Tate et al., 2022). Approximately half of adult mental disorders begin during adolescence (AlAzzam and Abuhammad, 2021), making these early years of life a key time at which to intervene to support good mental health, and to prevent or reduce later poor mental health outcomes. Secondary school students are in this relatively sensitive adolescent period full of academic demands, social interactions, and personal identity formation, making them a critical population for mental health research.

China, with 158 million adolescents aged 10–19 years according to the 2020 National Population Census, has seen a growing public concern in the mental health of adolescents. Over the past decades, China’s society has undergone dramatic changes with regard to industrialization and urbanization. The rapid socio-economic development and the competitive educational environment have added layers of complexity to the mental health landscape of adolescents. The unique sociocultural context, compounded by the rapid pace of economic development and societal transformation, presents distinct challenges and stressors to the mental well-being of young people in China. The mental health of adolescents in China is a multifaceted issue that requires the attention of policymakers, educators, healthcare providers, and families. There is an urgent need for more research to support the mental health of Chinese adolescents in these challenging times.

Previous studies have explored the mental health problems in adolescents and studied associated factors. A previous study of 2,837 adolescents using Mental Health Inventory of Middle School Students (MMHI-60) showed that the mental health problems increased from 2016 to 2020 (Wu et al., 2022). A survey of 12,096 high school students in Shandong, in 2018 and 2021, revealed an increase in psychological problem scores on the Strengths and Difficulties Questionnaire (Chen et al., 2022). Another study also using MMHI-60 showed that the positive rate of mental health problems among 15,055 high school students was 41.8%, with the most frequent mental health problem of academic stress (58.9%) (Luo et al., 2020). Higher grades, physical disease, chronic constipation, alcohol consumption, engagement in sexual behavior, residence on campus, and living in nonurban areas and with single-parent families were significantly associated with higher odds of having mental health problems (Luo et al., 2020). Another study demonstrated that gender, grade, region, and academic period and a two-parent family were associated with adolescents’ mental health (Han et al., 2023). Family socioeconomic status including family income, parental education level, and parental occupation, has a significant positive effect on mental health of adolescents in China (Yang et al., 2022). However, most studies have focused on a single dimension of mental health such as depression or anxiety symptoms, while more comprehensive surveys covering multiple dimensions are rare.

In the present study, we aimed to provide a comprehensive assessment of the mental health status among secondary school students in Shunde District, Guangdong, by employing a battery of well-validated scales, including Mental Health Inventory of Middle School Students (MMHI-60), School Refusal Behavior Evaluation Scale (SRBES), School Refusal Assessment Scale for Children (SRAS-C), Revised Chinese Internet Addiction Scale (CIAS-R), Pittsburgh Sleep Quality Index (PSQI), Adolescent Suicide Tendency Scale (ASTS), Connor-Davidson Resilience Scale (CD-RISC) and Self-esteem scale (SES). These scales cover a range of mental health aspects, including general mental health issues, school refusal behavior, Internet addiction, sleep quality, suicidal tendencies, resilience, and self-esteem. Understanding the prevalence of mental health issues and identifying associated factors are essential for developing targeted interventions and policies to support the mental well-being of this vulnerable population. Our study will serve as a valuable reference for mental health promotion, mental illness prevention, and policymaking across China.

## Methods

### Study design and participants

This is an observational study of Chinese secondary school children from all the five secondary schools in Shunde District, Guangdong Province via an on-line survey system from January, 2022 to December, 2023. The inclusion criteria included: (1) secondary school students; (2) able to provide informed consent; (3) able to complete the survey within a required time. The exclusion criteria included: children who dropped out of school or studied in special education schools. Multiple scales were used to evaluate a range of mental health aspects, including general mental health issues, school refusal behavior, internet addiction, sleep quality, suicidal tendencies, resilience, and self-esteem via questionnaire survey. Meanwhile, sociodemographic characteristics were collected including name, sex, age, grade, resident place, siblings, boarding, economic status, grade ranking and parents’ highest educational attainment. The economic status was categorized as poor, below average, average, and rich. These categories were defined based on per capita disposable income relative to the average per capita disposable income in Shunde. Specifically, poor was defined as per capita disposable income less than 0.5 times the average per capita disposable income in Shunde; below average was defined as per capita disposable income greater than or equal to 0.5 times but less than the average per capita disposable income in Shunde; average was defined as per capita disposable income greater than or equal to the average but less than twice the average per capita disposable income in Shunde; and rich was defined as per capita disposable income greater than or equal to twice the average per capita disposable income in Shunde. The grade ranking was defined as the top one-third, middle one-third, and bottom one-third according to the ranking of the total exam scores.

Data collection was conducted on a class-by-class basis. With students filling out questionnaires directly on computers in the school computer lab through the on-line survey system. Researchers were provided with unified training and were given the explanation of the questions in the questionnaire to ensure that the respondents had a correct and consistent understanding of the questionnaire. Moreover, instructions were provided to guide students when they were filling out the questionnaires to ensure the quality of the study. Incomplete questionnaires would be excluded from the analysis. In order to minimize response bias, we took several actions including that the on-line survey system was anonymous and all data were de-identified. Additionally, before the participants responded to the on-line survey, we ensured thorough communication so that participants felt no psychological pressure when completing the questionnaire.

This study was conducted with IRB approval at The Fourth People’s Hospital of Shunde (Shunde Wu Zhongpei Memorial Hospital) (Number: 2022-2). All participants provided their informed consent. The study followed the declaration of Helsinki guidelines.

### MMHI-60

MMHI-60 is a validated and widely used self-report measure of mental health problems. The scale consists of 10 subscales, including obsessive-compulsive symptoms, paranoid ideation, hostility, interpersonal sensitivity, depression, anxiety, academic stress, maladaptation, emotional, disturbance and psychological imbalance. Each subscale contains 6 items, totaling 60 items. A 5-point Likert scale is used for scoring, with 1 to 5 indicating “never,” “mild,” “moderate,” “quite a bit” and “severe,” respectively. The total score is calculated by summing the scores of all items and dividing by 60. A total score of 2 or above is considered abnormal in mental health screening. A total score of 2 to 2.99 indicates mild mental health issues, 3 to 3.99 indicates moderate mental health issues, 4 to 4.99 indicates more severe mental health issues, and 5 indicates very severe mental health issues.

### School refusal behavior scale for children and school refusal assessment scale for children

The School Refusal Behavior Scale for Children (SRBES) is a questionnaire developed by domestic scholars, including Chen Yuxiao and others, which is suitable for assessing school refusal behavior in Chinese children. It includes 19 items divided into five factors: defiant behavior, school alienation, negative emotions, learning ability, and physical sensations. The items are rated on a 5-point scale, with 1 to 5 points representing “completely disagree,” “partially disagree,” “uncertain,” “partially agree” and “completely agree,” respectively. The total score of the questionnaire ranges from 19 points (the lowest degree of school refusal behavior) to 95 points (the highest degree of school refusal behavior) and those scoring above 57 points are considered to exhibit school refusal behavior.

The School Refusal Assessment Scale for Children (SRAS-C) originated from the School Refusal Assessment Scale (SRAS) developed by Kearney and Silverman in 1993, which was revised in 2002. The revised scale is divided into child and parent versions, each with 24 items, rated on a 0 to 6 seven-point scale. Both versions are divided into four factors: fear and negative emotions caused by school, avoidance of unpleasant social interactions and specific situations, seeking attention from significant others, and seeking stimulation outside of school, with each factor containing 6 items. The scale investigates the specific reasons for school refusal among children and adolescents and has been proven to have good reliability and validity.

### Revised Chinese internet addiction scale

Internet addiction was measured by the CIAS-R. The scale consists of 26 items, composed of two subscales: “Core Symptoms of Internet Addiction” and “Internet Addiction Related Issues.” The “Core Symptoms of Internet Addiction” subscale includes three factors: compulsive internet use, withdrawal symptoms, and tolerance. The “Internet Addiction Related Issues” subscale includes two factors: interpersonal and health issues, and time management issues. A 4-point rating system is used (1 point = strongly disagree, 2 points = disagree, 3 points = agree, 4 points = strongly agree), with the scores of the items included in each subscale added together to obtain the subscale score, and the sum of the subscale scores is the total score. The higher the total score, the greater the likelihood of internet addiction and individuals scoring in the top 5% were considered as high-risk groups according to the previous study (Chen et al., 2003).

### PSQI

The questionnaire is primarily used to assess the overall sleep quality of the subjects. The questionnaire is widely used as a measure of sleep quality in China (Zhou et al., 2020; Zhou et al., 2022). The scale consists of 19 self-rated items, which are categorized into 7 components: subjective sleep quality, sleep latency, sleep duration, sleep efficiency, sleep disorders, sleep medication, and daytime dysfunction. The total score ranges from 0 to 21, with a higher total score indicating poorer sleep quality.

### Adolescent suicide tendency scale

The Adolescent Suicide Tendency Scale is designed to assess suicidal tendencies, with evaluation factors including concealment, despair, identification with suicide, and suicide preparedness. It consists of 20 items, of which there are 17 positively worded items and 3 reverse-worded items (Zhou et al., 2022; Huang et al., 2023; Di Vincenzo et al., 2024). The scoring range is from 0 to 4 points. There are 4 subscales, covering “Concealment,” “Despair,” “Identification with Suicide,” and “Preparation for Suicide.” The total score is the sum of all item scores, with a range between 0 and 60 points. The assessment results are divided into 5 levels: 0 to 14 is “Normal,” 15 to 21 is “Mild,” 22 to 30 is “Moderate,” 31 to 40 is “Moderately severe,” and above 41 is “Severe.” The total score of 15 or above in considered abnormal.

### Connor-Davidson resilience scale

The scale was revised by Xiao Nan from the Chinese University of Hong Kong in 2007 based on the Connor-Davidson Resilience Scale. The scale consists of 25 items and includes three dimensions: resilience, strength, and optimism. The 5-point Likert scale was used, ranging from 0 (not at all) to 4 (very much), with a total score of 0 to 100 points. Higher total scores indicate higher psychological resilience.

### Self-esteem scale

The SES was developed by Rosenberg in 1965 and was initially designed to assess adolescents’ overall feelings about self-worth and self-acceptance. The scale consists of 10 items and uses a four-point rating system, ranging from 1 (strongly agree) to 4 (strongly disagree). The total score ranges from 10 to 40 points, with higher scores indicating a higher level of self-esteem.

### Statistical analysis

Continuous variables were described using mean±SD and categorical variables were described with numbers (percentages). Difference of continuous variables between two groups were compared using student’s *t* test and difference of categorical variables between groups were compared with chi-square test. The correlation between the scores of difference scales were tested by Pearson correlation. Univariable linear regression and multi-variable linear regression was used to explore the potential associated factors for each scale. Variance inflation factor (VIF) was used to evaluate the multicollinearity between covariates. All data were analyzed by R (3.6.2) and all *p* values < 0.05 was considered as significant.

## Results

### Baseline characteristics

A total of 8,013 secondary school children from all the 5 secondary schools were included in the present study. The baseline characteristics of the students are demonstrated in Table 1. In brief, 4,405 students (55.0%) were boys and the mean age was 14.5 ± 1.8 years old. There were more middle school students (5,438, 67.1%) than high school students (2,665, 32.9%), with the numbers of students from grade 7 to grade 12 of 2,438 (30.4%), 1,788 (22.3%), 1,213 (15.1%), 847 (10.6%), 890 (11.1%) and 837 (10.4%), respectively. Most students were locals (4,995, 62.3%) and resident students (6,194, 77.3%). 1,494 (18.6%) students had siblings. The economic status of family for most students (7,272, 89.7%) were at average level. The highest educational attainment for most fathers and most mothers were middle school (2,822, 35.2% and 2,842, 35.5%, respectively). 3,079 (38.4%) students moved away from their hometowns.

**Table 1:** Baseline characteristics.

Variable	Overall
	(*N* = 8,013)
Gender	
Female	3,608 (45.0%)
Male	4,405 (55.0%)
Age	
Mean (SD)	14.5 (1.78)
Grade	
Grade 7	2,438 (30.4%)
Grade 8	1788 (22.3%)
Grade 9	1,213 (15.1%)
Grade 10	847 (10.6%)
Grade 11	890 (11.1%)
Grade 12	837 (10.4%)
Registration location	
Non-local	3,018 (37.7%)
Local	4,995 (62.3%)
Only child	
No	6,519 (81.4%)
Yes	1,494 (18.6%)
Boarding	
No	1819 (22.7%)
Yes	6,194 (77.3%)
Economic status	
Poor	144 (1.8%)
Below average	1,271 (15.9%)
Average	6,201 (77.4%)
Rich	397 (5.0%)
Father’s educational attainment	
Primary school	554 (6.9%)
Junior high school	2,822 (35.2%)
High school	2,527 (31.5%)
Associate degree	976 (12.2%)
Undergraduate	990 (12.4%)
Master’s degree	98 (1.2%)
Ph.D.	46 (0.6%)
Mother’s educational attainment	
Primary school	908 (11.3%)
Junior high school	2,842 (35.5%)
High school	2,268 (28.3%)
Associate degree	1,023 (12.8%)
Undergraduate	877 (10.9%)
Master’s degree	59 (0.7%)
Ph.D.	36 (0.4%)
Away from hometown	
No	4,934 (61.6%)
Yes	3,079 (38.4%)

### Overall profiles for the scales

We first demonstrated the overall scores and abnormal percentages for each scale (Table 2). 927 (11.6%) students reported having engaged in self-harming behavior. The mean scores for SRBES were 42.9 ± 15.9 and there were 1,575 (19.7%) students with SRBES scores above 57. The mean scores for ASAT were 8.98 ± 9.51 and the numbers of students showing mild, moderate, moderately severe to severe suicide tendency were 753 (9.4%), 517 (6.5%), 250 (3.1%) and 103 (1.3%), respectively. 435 (5.4%) students had abnormal CIAS scores. 1,575 (19.7%) students had school refusal behavior. 103 (1.3%) students had severe suicide tendency.

**Table 2:** The overall profiles for the scales.

Scale	Overall
	(*N* = 8,013)
MMHI-60	
Mean (SD)	1.63 (0.588)
Normal	6,216 (77.6%)
Mild	1,508 (18.8%)
Moderate	262 (3.3%)
Moderately severe	24 (0.3%)
Severe	3 (0.0%)
SRBES	
Mean (SD)	42.9 (15.9)
Abnormal	1,575 (19.7%)
Normal	6,438 (80.3%)
SRAS-C	
Mean (SD)	1.93 (0.878)
CIAS-R	
Mean (SD)	36.5 (11.6)
Abnormal	435 (5.4%)
Normal	7,578 (94.6%)
PSQI	
Mean (SD)	8.44 (3.48)
ASTS	
Mean (SD)	8.98 (9.51)
Normal	6,390 (79.7%)
Mild	753 (9.4%)
Moderate	517 (6.5%)
Moderately severe	250 (3.1%)
Severe	103 (1.3%)
CD-RISC	
Mean (SD)	57.7 (19.1)
Median [Min, Max]	57.0 [0, 110]
SES	
Mean (SD)	28.3 (4.77)
Self harm	
No	7,086 (88.4%)
Yes	927 (11.6%)

**Figure 1:**
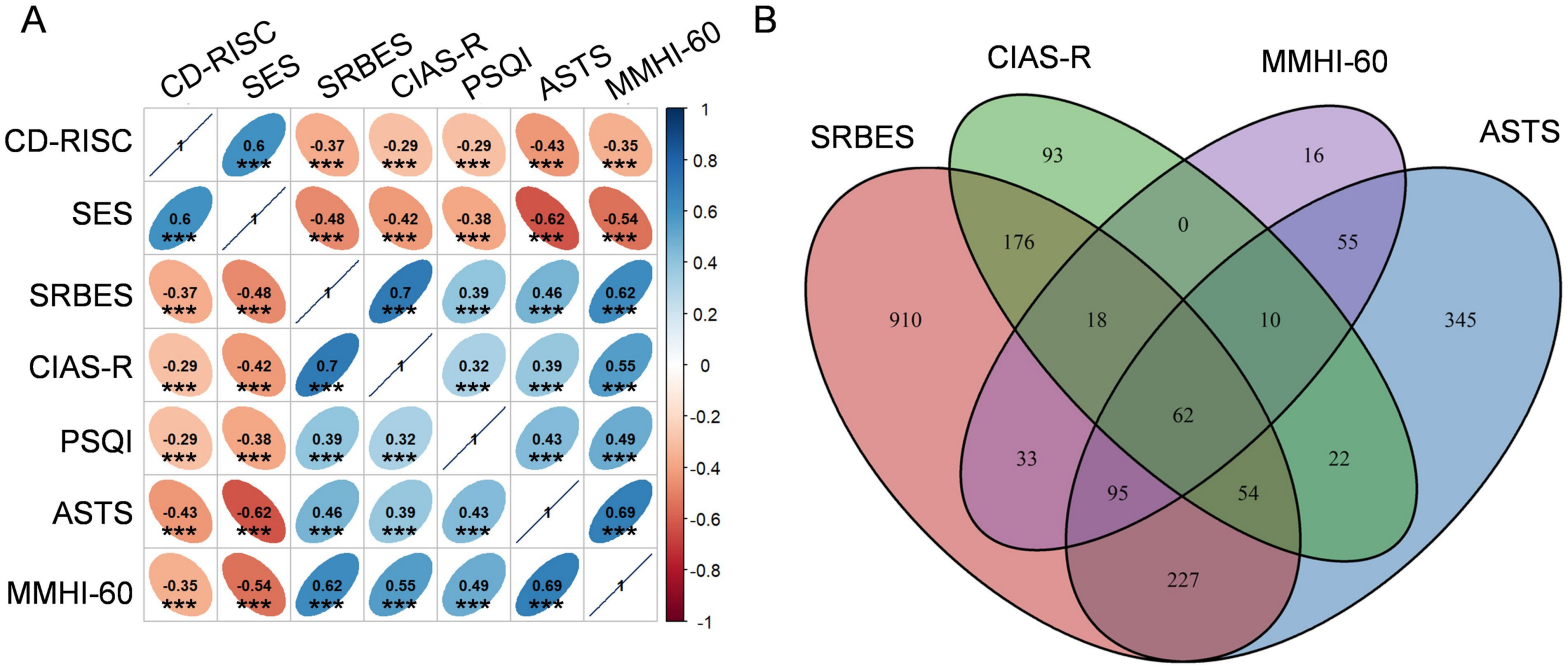
Overall profiles for the scales. **(A)** Correlation between different scale scores. Numbers in the cells indicate the spearman coefficients. ***p < 0.001. **(B)** Overlap between the abnormal students indicated by different scales.

We then explored the correlation between these scales (Figure 1A). The scores of RISC and SES were positively correlated with each other, and were negatively correlated with the scores of SRBES, CIAS, PSQI, ASTS and MMHI-60. The scores of SRBES, CIAS, PSQI, ASTS, and MMHI-60 were positively correlated with each other. These results suggested that the scores of these scales are reliable since the higher scores of RISC and SES and the lower scores of SRBES, CIAS, PSQI, ASTS, and MMHI-60, the better mental health is. We then demonstrated the overlap between the abnormal students indicated by SRBES, CIAS, MMHI and ASTS. There were 62 students showing abnormal behavioral or emotional problems suggested by all the above four scales (Figure 1B), who needed timely intervention including mental health education and psychological counseling.

### Associated factors for mental health

We then explored the associated factors of mental status including general mental health issues, school refusal behavior, Internet addiction, sleep quality, suicidal tendencies, resilience, and self-esteem. Higher age, higher grade, boarding status, lower grade ranking, worse economic status, being away from hometown and self-harm behaviors were associated with worse general mental health status, as indicated by higher MMHI-60 scores (*p* < 0.05, Table 3). In contrast, male, having a non-local registration location and having higher parents’ educational attainment were associated with lower MMHI-60 scores (*p* < 0.05, Table 3). We further performed VIF to evaluate the multicollinearity between covariates. The variables age and grade had high multicollinearity with VIF values higher than 10 (Supplementary Table S1), thus grade was not further included in the multivariable linear regression. In contrast, the rest variables had mild to moderate multicollinearity and did not severely impact the regression model, with the VIF values ranging from 1.014283 to 1.966796 (Supplementary Table S1). In the multivariable linear regression, only age, sex, grade ranking, economic status, father’s educational attainment and self-harm behavior were associated with MMHI-60 scores (*p* < 0.05, Supplementary Table S2).

**Table 3:** Association between sociodemographic factors and MMHI-60 scores.

Variable	Description	β-coefficient	95% CI Lower limit	95% CI Upper limit	*P* value
MMHI-60					
Age	Continuous variable	0.079389	0.07237	0.08641	1.15E-105
Gender	Male vs. female	−0.137332	−0.1631	−0.1116	1.79E-25
Grade	Continuous variable	0.086688	0.07936	0.09402	4.30E-115
Registration location	Non-local vs. local	−0.04635	−0.0729	−0.0198	6.30E-04
Only child	Yes vs. no	−0.030802	−0.0639	0.00227	6.79E-02
Boarding	Yes vs. no	0.056198	0.02547	0.08693	3.39E-04
Grade ranking	Ordinal variable from top to bottom ranking	0.092686	0.07413	0.11124	1.63E-22
Economic status	Ordinal variable from rich to poor	0.173794	0.14877	0.19882	9.76E-42
Father’s educational attainment	Ordinal variable from primary school to PhD	−0.029472	−0.0402	−0.0188	7.00E-08
Mother’s educational attainment	Ordinal variable from primary school to PhD	−0.036454	−0.047	−0.0259	1.55E-11
Away from hometown	Yes vs. no	0.038679	0.07237	0.08641	4.19E-03
Self-harm behavior	Yes vs. no	0.765983	−0.1631	−0.1116	0.00E+00

Similar results were observed for school refusal behavior using the SRBES and SRAS-C that the higher age, higher grade, boarding, lower grade ranking, worse economic status, away from hometown and self-harm behaviors were associated with more school refusal behavior, while male, non-local registration location, only child and higher parents’ educational attainment were associated with less school refusal behavior (*p* < 0.05, Supplementary Table S3).

We further studied the associated factors for Internet addiction by CIAS-R, sleep quality by PSQI, suicidal tendency indicated by ASAT, resilience by CD-RISC and self-esteem by SES and observed similar results except that no association was observed between PSQI and age/only child, ASAT and registration location/away from hometown, CD-RISC and registration location and SES and boarding (Supplementary Table S3). Altogether, these results suggested that the above variables are important factors associated with the mental health of the secondary school students.

### Gender and mental health status

Based on the above results, we then explored the differences of these scales between boys and girls. Girls had significantly higher scores of MMHI-60, SRBES, SRAS-C, PSQI, ASTS, and CIAS-R but significantly lower scores of SES and CD-RISC than boys (*p* < 0.05, Table 4), indicating that the mental health problems were more common in girls. In consistent, there were more abnormal students indicated by MMHI-60, SRBES, ASTS, and CIAS-R in girls than boys (*p* < 0.05, Table 4).

**Table 4:** Comparison of the scales between girls and boys.

Scale	Girls	Boys	*P* value
	(*N* = 3,608)	(*N* = 4,405)	
Self-harm behaviors			
No	3,147 (87.2%)	3,939 (89.4%)	0.002
Yes	461 (12.8%)	466 (10.6%)	
MMHI-60			
Mean (SD)	1.71 (0.604)	1.57 (0.567)	<0.001
Normal	2,655 (73.6%)	3,561 (80.8%)	<0.001
Mild	791 (21.9%)	717 (16.3%)	
Moderate	149 (4.1%)	113 (2.6%)	
Moderately severe	13 (0.4%)	11 (0.2%)	
Severe	0 (0%)	3 (0.1%)	
SRBES			
Mean (SD)	44.3 (15.1)	41.8 (16.4)	<0.001
Abnormal	745 (20.6%)	830 (18.8%)	0.046
Normal	2,863 (79.4%)	3,575 (81.2%)	
SRAS-C			
Mean (SD)	2.01 (0.808)	1.86 (0.931)	<0.001
CIAS-R			
Mean (SD)	37.0 (11.1)	36.0 (12.0)	<0.001
Abnormal	173 (4.8%)	262 (5.9%)	0.03
Normal	3,435 (95.2%)	4,143 (94.1%)	
PSQI			
Mean (SD)	8.91 (3.42)	8.05 (3.49)	<0.001
ASTS			
Mean (SD)	10.2 (10.1)	8.01 (8.86)	<0.001
Normal	2,705 (75.0%)	3,685 (83.7%)	<0.001
Mild	413 (11.4%)	340 (7.7%)	
Moderate	276 (7.6%)	241 (5.5%)	
Moderately severe	154 (4.3%)	96 (2.2%)	
Severe	60 (1.7%)	43 (1.0%)	
CD-RISC			
Mean (SD)	54.8 (17.3)	60.1 (20.2)	<0.001
SES			
Mean (SD)	27.9 (4.87)	28.7 (4.67)	<0.001

**Figure 2:**
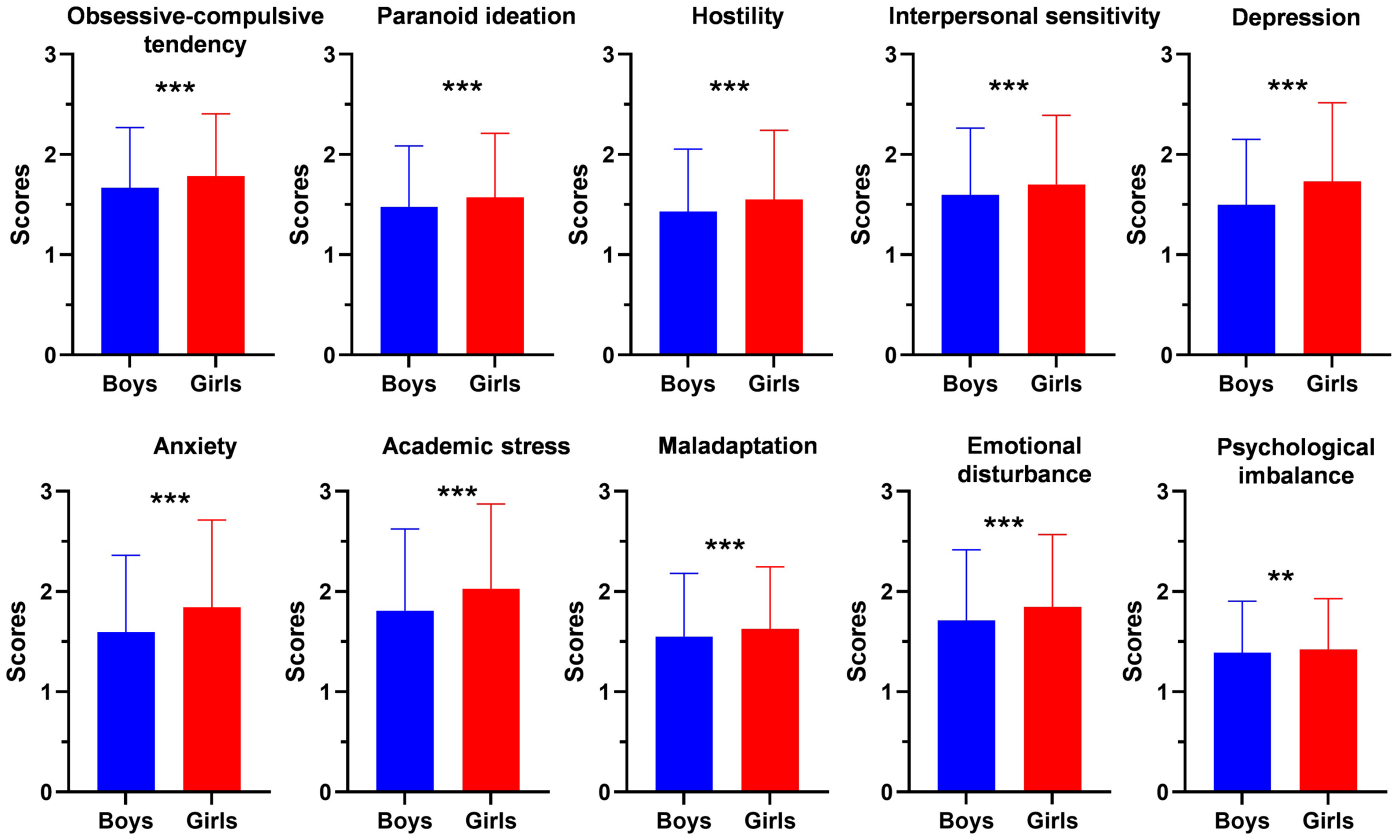
Comparison of the MMHI-60 subscales between girls and boys including obsessive-compulsive tendency, paranoid ideation, hostility, interpersonal sensitivity, depression, anxiety, academic stress, maladaptation, emotional disturbance and psychological imbalance. Error bars indicate the standard deviation. **p < 0.01; ***p < 0.001.

We also studied the differences of the subscales of MMHI-60 between boys and girls including obsessive-compulsive tendency, paranoid ideation, hostility, interpersonal sensitivity, depression, anxiety, academic stress, maladaptation, emotional disturbance and psychological imbalance. The scores of all the subscales were higher in girls than boys (*p* < 0.05, Figure 2). All these results suggested that girls are more vulnerable to mental health problems and need more attention.

## Discussion

The present study used multiple scales to assess the mental health status in secondary school students from China, providing a comprehensive insight into the prevalence and associated factors of the mental health problems in this vulnerable population. The prevalence of abnormal mental health was 22.4% in MMHI-60, 19.7% in SRBES, 5.4% in CIAS-R and 20.3% in ASTS, respectively. Better economic status, higher father’s education, higher mother’s education and higher grade ranking were associated with fewer mental health issues. A higher incidence of mental health problems was observed in girls than boys. These findings highlight the significant prevalence of mental health issues and the importance of understanding the multifaceted nature of these problems in adolescents. To our knowledge, this is the first study that uses comprehensive scales to evaluate the mental status including general mental health issues, school refusal behavior, Internet addiction, sleep quality, suicidal tendencies, resilience, and self-esteem in secondary school students.

The study revealed that a considerable proportion of students exhibited signs of mental health issues as indicated by various scales. Notably, the MMHI-60 showed an abnormal mental health prevalence of 22.4%. The high prevalence of school refusal behavior (19.7% as per SRBES) and Internet addiction (5.4% as per CIAS-R) further underscores the need for targeted interventions. A cross-sectional study in Henan Province, China, reported a positive rate of mental health problems among high school students at 41.8% indicated by the MMHI-60 (Huang et al., 2023). It has been reported that approximately 2–5% of all school-aged children and adolescents experience school refusal behavior (Di Vincenzo et al., 2024). The prevalence of Internet addition has been reported to be 13.4% among 7,990 vocational high school students (Gao et al., 2022). Another cross-sectional study conducted among Chinese adolescents showed a prevalence of internet addiction at 12.8% (Fan et al., 2023). Even though the prevalence of the above mental health problems were varied between the above studies, all these findings suggested the severe situation of mental health problems in secondary school students. Moreover, a total of 62 students (0.8%) in the present study showing abnormal mental problems suggested by all the four scales of SRBES, CIAS, MMHI, and ASTS. These students may be experiencing severe mental health problems and need immediate targeted interventions.

Exploring the associated factors of mental health problems for adolescents may provide useful information for understanding the development of mental health status and shed light on the prevention of poor mental health. In the present study, gender, socioeconomic status, parental education, and grade rank were found to be significantly associated with mental health outcomes indicated by multiple scales. Students from lower socioeconomic backgrounds and those with parents having lower educational attainment exhibited poorer mental health scores. As supported by previous studies, risks of occurrence of poor mental health are associated with numerous social-demographics factors, such as gender (Campbell et al., 2021), family economic status (Lin and Guo, 2024), parental marital status (Lindstrom and Rosvall, 2016), or family history of mental disorders. The association between the above associated factors and mental health were generally consistent in different aspects of mental health including general mental health issues, school refusal behavior, Internet addiction, sleep quality, suicidal tendencies, resilience, and self-esteem, suggesting the important role of these factors. These findings underscore the complex interplay between various associated factors and mental health, highlighting the importance of a multifaceted approach to prevention and intervention, including support from family and school environments, as well as the importance of promoting healthy coping strategies and self-esteem in adolescents.

Among these associated factors, we observed significant difference in mental health between girls and boys, with girls showing significantly higher scores in scales measuring mental health, school refusal behavior, sleep disturbances, suicidal tendencies, and Internet addiction. These results were supported by another cross-national investigation of adolescents across 73 countries reported that the gender gap in mental health is largely ubiquitous, with girls consistently having worse mental health on average (Campbell et al., 2021). The higher prevalence of mental health problems in girls may be contributed by multiple factors including the earlier onset of puberty, increased pressure to conform to societal gender norms, higher sensitivity to peer relationship in girls and etc. (Yoon et al., 2023). The cultural background of China may also contribute to the difference of mental health between girls and boys. In China, college entrance and senior high school entrance examinations decide whether students can enter a key university or senior high school. Academic pressure has become common source of mental health problems in Chinese adolescents. Girls have shown to have higher academic pressure (Zhang et al., 2019), which may result in higher prevalence of mental health problems. Girls may face additional pressures related to balancing academic performance with other expectations, such as maintaining a certain image or fulfilling traditional gender roles. Traditional gender roles often place a heavy emphasis on appearance, behavior and emotional expression for girls. The findings emphasize that more attention should be given to girls and it is crucial to develop policies that address the gender-specific vulnerabilities.

Despite the increasing prevalence of mental health problems among adolescents, mental health services remain underfunded and under-resourced in China (Chen et al., 2024). China has a large gap between the burden of mental health problems and the capacity of mental health services for adolescents. Current policies do not comprehensively address the multifaceted nature of mental health problems. More comprehensive strategies based on the current findings are needed, which require a whole-of-government approach, not only to increase family income, but also to involve a wider community including adolescents, and their families.

While the study provides valuable insights, it is not without limitations. First, the cross-sectional design limits the ability to establish causal relationships. The current study lacks the longitudinal data to assess long-term outcomes of the mental health issues. Future research should employ longitudinal studies to better understand the development of mental health issues over time. Second, the current study only collected sociodemographic characteristics including name, sex, age, grade, resident place, siblings, boarding, economic status, grade ranking and parents’ highest educational attainment to study their association with the mental health issues. Other environmental factors (e.g., parental conflict, academic pressure) or genetic factors were not studied, which may also influence mental health problems. Third, the present study only focused on a specific region in China, which may limit the generalizability of the findings to rural China or other provinces. Future studies should aim to include diverse populations to capture a broader perspective on adolescent mental health. Fourth, we identified a total of 62 students (0.8%) in the present study showing abnormal mental problems suggested by all the four scales of SRBES, CIAS, MMHI and ASTS. The small sample size may limit the precision of risk estimation. We also lack further longitudinal data to further study the development of mental health problems for these students. Fifth, the present study was conducted during the COVID-19 pandemic. Previous studies have demonstrated the influence of COVID-19 pandemic on the mental health (Wang et al., 2021; Sicouri et al., 2023). However, the present study did not further explore the influence of COVID-19 pandemic since Shunde District is less influenced by the COVID-19 pandemic during the study period.

In conclusion, the study presents a comprehensive picture of the mental health landscape among secondary school students, highlighting the need for targeted interventions and policy changes to support the mental well-being of this vulnerable population. By addressing the identified associated factors and promoting resilience and self-esteem, it is possible to mitigate the impact of mental health issues and foster a healthier future for these students.

## Data Availability

The raw data supporting the conclusions of this article will be made available by the authors, without undue reservation.
